# The British Association

**Published:** 1893-09-23

**Authors:** 


### The Hospital

AN INSTITUTIONAL JOURNAL OF

Science, Medicine, Nursing, and Philanthropy.

*For Hospitals, Asylums, Infirmaries, Dispensaries, Medical Practitioners, Students, and Nurses.*

VOL. XIV.—No. 365.] [SEPT. 23, 1893.

### THE BRITISH ASSOCIATION.

It is the fashion for those persons who are not invited to take part in the work of the British Association to rail upon those who are, and to make second-rate jokes at the expense of "Peripatetic Science." But science and its votaries are something like the stage and its players: they may do well at private rehearsals; they only do their worthiest and best in the presence of a popular and sympathetic audience. Many scientists will resent this; but they will usually be found to be of the second-rate order. Those men of science who in addition to their capacity for research have the faculty of clear and philosophical exposition, always take pleasure in proclaiming *urbi et orbe* the facts and truths which, though discovered by the methods of science, are the true and legitimate property of the whole world.

Since its foundation the British Association has had for its presidents at various times men of whom the whole world has declared that they were meritorious and distinguished in the highest possible degree. Never has there been a president of whom it might with more truth be said than of Dr. Burdon Sanderson, that in him science has a son whose loyalty is sincere, whose capacity is great, and whose achievements have carried his name to the remotest bounds of scientific activity and success.

Dr. Burdon Sanderson is a physiologist, but not the kind of physiologist who merely makes experiments and accumulates more or less doubtful facts. He is a man who sees at least this much, that physiology is not and never can be the measure of the world. He sees even more than this, that physiology itself is one thing, and that his own knowledge of it is another, and possibly sometimes quite a different thing. Professor Sanderson is proud to have been a young man, but he is prouder still now to be an older one. He knew far more things forty years ago, at least in his then estimation, than he knows to-day. But what he knows to-day he knows with much more satisfaction and intelligence; since he knows how much of his knowledge is real and actual, and how much is hypothetical and doubtful.

The history of physiology is profoundly interesting. It is a history, not of facts alone, but of persons, and of persons much more than facts. Professor Sanderson dates from Treviranus. Treviranus worked at the beginning of the present century; and he set himself upon a task which he perceived would occupy his whole life. This was no other than the mastery of the knowledge of life and all living things. This task, so soon as its nature was propounded to the young men of that generation, dazzled and charmed them beyond compare. The secret of life had as much fascination for the young scientists of the beginning of the century, as the secret of the elixir of life has had for alchemists and the lovers of mystery in all ages. In no long time the secret was declared to stand revealed, and the mystery to have been elucidated. Life was the product of mechanism. The human body, the animal body, the tree, and the vegetable was each a mechanism. It was a mechanism which could be taken to pieces and demonstrated in every part. It was a mechanism which, since science had analysed it, science would doubtless in due time synthetically recompose. The arch-magician who had pursued the secret to its last recesses was Schwann and the discovery which rewarded the patient and intrepid explorer was the discovery of the cell. Every organism—human, animal, vegetable—was composed of cells; and all that was needed for the creation of life, and of newer and ever more varied forms of life on this planet, was the synthesis of cells in their due and scientific order. Here was a discovery of science so glorious and far reaching that it might well displace from their balance the strongest intellects. Many such intellects did lose their balance, and believed with a faith that was truly apostolic that the morning of a new creation was about to dawn. Professor Burdon Sanderson himself was probably in his young days a disciple of the new scientific cult.

But let us, with the freedom of the ancient story-teller, leap over a period of forty years. The ardent student of physiology of Edinburgh University has become the grave and reverend president of the British Association for the promotion of Natural Science. He stands in the midst of toilers whose whitening hair tells of years of labour and thought. Many of those toilers remember the ardour and hopefulness of their youth; they remember also the sober and modest realities which have taken the place of the wondrous visions of a new creation which stirred the enthusiasm of their boyhood. Dr. Burdon Sanderson sets before them the conclusions which occupy his mind at sixty as well as those which filled his imagination at sixteen. Is life a product of mechanism now? The President of the British Association confesses that he does not know. What is life? This leader of all the modern scientists cannot tell. He admits with the materialists of the early part of the century that "protoplasm is the basis of life"; but he affirms, what many other materialists will not thank him for affirming, that in his judgment "life is also the basis of protoplasm."

This is truly a marvellous result of the scientific industry of the past half century. The wonder is, not that men of science have failed to find out the true and veritable nature of life; but that they know they have failed and willingly admit the fact. Dr. Burdon Sanderson has a new name, or rather an old name refurbished, which he thinks may do duty for all those phenomena which are associated with life, and by virtue of which every organism, organ, and cell is enabled to play its part in the world of living things. This name is "specific energy." Few will be found to quarrel with it. That it is philosophical most thinkers will admit. It affirms a fact; it does not affirm a theory of life and living things. It need not be further considered here than to point out that it shows us the fair, candid and impartial attitude of mind of the scientific leaders of the present day.

Medical men, like other Britons, are a practical race, and they not unnaturally ask what of a practical nature are physiologists like Professor Burdon Sanderson doing for the art of medicine and for the service of common mankind? The answer to this question is definite and convincing to those who understand. Physiologists deserve the sympathy, and even the admiration, of the world in their patient, unwearied, and often heroic efforts to wrest from a too reluctant Nature the practical knowledge which will enable the physician and the surgeon to prevent and to cure disease with unfailing certainty. Professor Sanderson briefly sketched the work of the past and the present generations. It seemed to lovers of knowledge almost pathetic to hear the learned physiologist complain of the lack of recognition on the part of those in authority. All the world takes advantage of medical knowledge when it is gained, but a part of the world covers with odium those who devote their lives to the gaining of it. These two attitudes of mind cannot both be right. Those who fight against the methods of physiology should resolutely refuse the aid of modern medicine, which is founded upon modern physiology, when overtaken by sickness and dangerous disease. We claim as we have claimed before, absolute freedom for the physiologist and all his ways. To his own master he must stand or fall. May we not go one step further and say that he who has discovered for us a new world of wonders and practical advantages is entitled to the honours of the explorer, and to the serious gratitude of every human being who profits, however little, by his arduous, enduring, and successful labours? If Governments were wise they would disregard the outcries of the unreasoning few, and make earnest and continuous efforts for the promotion of practical knowledge in every possible direction.

